# *Arabidopsis* HFR1 Is a Potential Nuclear Substrate Regulated by the *Xanthomonas* Type III Effector XopD_*Xcc*8004_

**DOI:** 10.1371/journal.pone.0117067

**Published:** 2015-02-03

**Authors:** Choon Meng Tan, Meng-Ying Li, Pei-Yun Yang, Shu Heng Chang, Yi-Ping Ho, Hong Lin, Wen-Ling Deng, Jun-Yi Yang

**Affiliations:** 1 Institute of Biochemistry, National ChungHsing University, Taichung, Taiwan; 2 Ph.D. Program in Microbial Genomics, National ChungHsing University and Academia Sinica, Taipei, Taiwan; 3 Department of Plant Pathology, National ChungHsing University, Taichung, Taiwan; 4 Institute of Biotechnology, National ChungHsing University, Taichung, Taiwan; 5 NCHU-UCD Plant and Food Biotechnology Center, National ChungHsing University, Taichung, Taiwan; 6 Agricultural Biotechnology Center, National ChungHsing University, Taichung, Taiwan; Shanghai Jiao Tong University, CHINA

## Abstract

XopD_*Xcc*8004_, a type III effector of *Xanthomonas campestris* pv. *campestris* (*Xcc*) 8004, is considered a shorter version of the XopD, which lacks the N-terminal domain. To understand the functions of XopD_*Xcc*8004_, *in planta*, a transgenic approach combined with inducible promoter to analyze the effects of XopD_*Xcc*8004_ in *Arabidopsis* was done. Here, the expression of XopD_*Xcc*8004_, in *Arabidopsis* elicited the accumulation of host defense-response genes. These molecular changes were dependent on salicylic acid and correlated with lesion-mimic phenotypes observed in *XVE::XopD_Xcc8004_* transgenic plants. Moreover, XopD_*Xcc*8004_ was able to desumoylate HFR1, a basic helix-loop-helix transcription factor involved in photomorphogenesis, through SUMO protease activity. Interestingly, the *hfr1-201* mutant increased the expression of host defense-response genes and displayed a resistance phenotype to *Xcc*8004. These data suggest that HFR1 is involved in plant innate immunity and is potentially regulated by XopD_*Xcc*8004_.

## Introduction

Post-translational modifications enable plants to rapidly modify the stability and activity of key factors that play fundamental roles in defense signaling during bacterial infections [1], [2]. Studies have shown that sumoylation, a reversible and dynamic process, appears to be essential for suppressing defense responses in non-infected plants [3]. The sumoylation machinery is composed of small ubiquitin-like modifier (SUMO)-specific E1 (SAE1/SAE2 heterodimer), E2 (SCE1), and E3 (SIZ1 and MMS21/HPY2) to make SUMO conjugates [4], [5]. Elevated accumulation of salicylic acid (SA) accompanied by localized programmed cell death in *sum1sum2* and *siz1*mutants exhibits increased resistance to *Pseudomonas syringae* and suggests that sumoylation machinery is likely to contribute to systemic-acquired resistance (SAR), resulting in enhanced resistance against further pathogen attacks [6–8].

The plant immune system is a multilayered type of immune response, which contains pathogen-associated molecular pattern-triggered immunity and effector-triggered immunity [9], [10]. To overcome the complex immune system, pathogens secrete or inject a range of effectors into host cells to manipulate host cellular functions and alter host defense responses [11], [12]. Although the functions of these virulence factors remain largely unknown, an increasing body of evidence demonstrates that pathogens employ a strategy to structurally or functionally mimic host cellular activities [13], [14]. In the past years, several bacterial effectors have been found to share structural similarity with SUMO proteases. Because bacteria do not have a SUMO system, it would be interesting to understand the role of pathogen effectors employing SUMO protease activity.

Previous studies have shown that the *Xanthomonas* type III effector XopD possesses desumoylation activity and localizes to nuclear foci in plant cells [15–17]. The subnuclear localization of XopD suggests that XopD may target SUMO-conjugated proteins in the plant nucleus. Indeed, XopD_*Xcc*B100_ from the strain B100 of *Xcc* specifically interacts with MYB30 to suppress its activity in activating plant defense responses required for anti-*Xcc* immunity [16]; XopD_*Xcv*85–10_ from the strain 85–10 of *Xanthomonas campestris* pv. *vesicatoria* (*Xcv*) specifically interacts with SIERF4 to suppress its activity in activating ethylene-induced responses, which is required for anti-*Xcv* immunity [18]. XopD is composed of an N-terminal domain, ERF-associated amphiphilic repression motifs, and a C-terminal SUMO protease domain [17], [19]. Although the C-terminal domain of XopD has SUMO peptidase and isopeptidase activities, lacking the functional N-terminal domain fails to suppress MYB30-mediated defense responses or desumoylation of SIERF4 [16], [18]. Thus, the N-terminus of XopD is essential for the virulence of *Xanthomonas in planta*. However, in the *Xcc*8004 strain, XopD_*Xcc*8004_ was considered as a XopD without N-terminal domain [17], [19]. Although XopD_*Xcc*8004_ has been shown to be able to be secreted via a *hrp*-dependent pathway and contains functional SUMO protease activity, the action of XopD_*Xcc*8004_ *in planta* is still largely unknown [19].

Recently, light has been considered as an important regulator in modulating plant immunity [20], [21]. The availability and quality of light affects the plant development, as well as influences the plant defense responses. For example, a high ratio of red to far-red light enhances plant resistance to herbivorous insects [22]; a low ratio of red to far-red light reduces plant resistance to bacterial pathogens [23], [24]. Thus, mutations in the photoreceptors greatly influence plant defense responses. In this study, an inducible expression system was used to study the functions of XopD_*Xcc*8004_ in transgenic *Arabidopsis* plants. Finally, we showed that HFR1, a basic helix-loop-helix transcription factor involved in light-signaling pathway, is a potential nuclear substrate regulated by XopD_*Xcc*8004_.

## Materials and Methods

### Plant materials and growth conditions

*Arabidopsis thaliana* was grown at 21°C under a 16-h light/8-h dark photoperiod for *Agrobacterium* transformations, and a 12-h light/12-h dark photoperiod for *Xanthomonas* spp. inoculations. *N*. *benthamiana* was grown at 26°C under a 16-h light/8-h dark photoperiod for *Agrobacterium* transient expression assay. The *Arabidopsis* WT, *hfr1–201* mutant, and *nahG* transgenic plants are in the Columbia ecotype background [6], [25].

### Plasmid constructions

*Xcc*8004 genomic DNA was used for amplification of the XC_1213 DNA fragment encoding XopD_*Xcc*8004_. An *A*. *thaliana* cDNA library was used for the amplification of the At1g02340 DNA fragment encoding HFR1. DNA fragments amplified by PCR using AccuPrime pfx DNA polymerase (Invitrogen) were subcloned into appropriate vectors by restriction site reconstructions. For the generation of *Arabidopsis* transgenic plants, PCR products were subcloned into the pER8 vector under the control of the XVE promoter [26]. For subcellular localization assays, PCR products were subcloned into pBA-YFP or pBA-CFP vectors under the control of the *Cauliflower mosaic virus* 35S promoter [27]. For yeast two-hybrid assays, PCR products were subcloned into pGADT7 and pGBKT7 vectors (Clontech) to generate AD-HFR1 and BK-XopD_*Xcc*8004_ constructs. For the expression of recombinant proteins, PCR products were subcloned into pET-SUMO (Invitrogen), pMAL-c2 (New England Biolabs), or pGEX4T-1 (GE Healthcare) vectors to produce N-terminal His-SUMO-, MBP-, or GST-tagged XopD_*Xcc*8004_, HFR1, or AS2 proteins. The K72A mutant of HFR1 and the C355A mutant of XopD_*Xcc*8004_ were generated by QuikChange site-directed mutagenesis (Stratagene) according to the manufacturer’s instructions. For homologous recombination, the 985-bp upstream and 976-bp downstream regions of *XopD*_*Xcc8004*_ were amplified from *Xcc*8004 genomic DNA and subcloned into a pK18mobsacB vector. To establish an *in vitro* sumoylation system, DNA fragments encoding *Arabidopsis* SAE1 (SAE1b), SAE2, and SCE1 were excised from the pACYCDuet-AtSAE1b-AtSAE2 and pCDFDuet-AtSUMO1(GG)-AtSCE1 plasmids [28], and subcloned into pET28a or pET29a vectors (Invitrogen) by restriction site reconstructions to produce His-tagged SAE1, SAE2, and SCE1 proteins. All plasmids were verified by DNA sequencing.

### *Arabidopsis* transformations

To obtain *Arabidopsis* transgenic plants, plasmids were introduced into the *Agrobacterium tumefaciens* strain ABI by the freeze-thaw method [29] and then transformed into *A*. *thaliana* Col-0 using the floral-dip method [30]. *Arabidopsis* seeds were grown on half-strength Murashige and Skoog (1/2× MS) medium containing hygromycin (12.5 μg mL^–1^) and carbenicillin (100 μg mL^–1^) to obtain transgenic lines. Homozygous seeds were further selected and amplified for analyses.

### Trypan blue staining

To characterize the lesion-mimic phenotype, *Arabidopsis* transgenic plants expressing XopD_*Xcc*8004_ were examined by trypan blue staining [31]. Briefly, transgenic seeds were germinated on 1/2× MS medium containing DMSO or 20 μM β-estradiol. Three-week-old seedlings were stained by boiling in a solution containing 10 mL of lactic acid, 10 mL of glycerol, 10 g of phenol, and 10 mg of trypan blue (dissolved in 10 mL of distilled water), and further destained in 2.5 g mL^–1^ chloral hydrate solution. Images were collected with a Leica ZM75 microscope.

### qRT-PCR

To measure *Arabidopsis* gene expression levels, total RNA was extracted by using the Trizol reagent and reverse transcribed into cDNAs using a Superscript III first-strand synthesis supermix according to the manufacturer’s instructions (Invitrogen). The qRT-PCR reactions were performed on an Eco real-time PCR system (Illumina) with the KAPA SYBR fast qPCR kit (Kapa Biosystems). Relative amounts of transcripts were normalized to the transcript level of a house keeping gene, *EF1α*. Experiments were repeated at least 3 times.

### Recombinant protein purifications and antibody production

To produce recombinant proteins, all constructs were transformed into *Escherichia coli* BL21 (DE3) cells and cultured at 24°C until the optical density at 600 nm reached 0.4. Then, isopropyl β-D-1-thiogalactopyranoside was added to a final concentration of 0.2 mM and cells were further incubated overnight. After cell lysis, bacterial cell extracts were purified using appropriate resins according to the manufacturer’s instruction. For *in vitro* sumoylation assays, *Arabidopsis* SAE1, SAE2, SCE1, and AtSUMO1 (with Gly-Gly at the C-terminus) proteins were purified using Ni^2+^-NTA resin (Qiagen). For *in vitro* pull-down assays, MBP- and GST-tagged proteins were purified using an amylose resin (New England Biolabs) and glutathione-Sepharose 4B (GE Healthcare), respectively. To generate a specific antibody against XopD_*Xcc*8004_, the His-SUMO-XopD_*Xcc*8004_ protein was purified using a Ni^2+^-NTA resin and cleaved with Ubl-specific protease 1 (Ulp1) to remove the His-SUMO tag. After cleavage, proteins were purified by using a Sephacryl S-200 HR gel filtration column (GE Healthcare) to obtain the XopD_*Xcc*8004_ protein alone. Finally, a rabbit polyclonal antibody raised against XopD_*Xcc*8004_ was obtained by affinity purification using a polyvinylidene difluoride membrane as a coupling matrix [32].

### Bacterial strains and inoculations

The *Xcc*8004 *ΔXopD* mutant strain was obtained using the sacB system [33]. Plasmid for homologous recombination was introduced into *Xcc*8004, and deletion mutant was verified by PCR. For bacterial inoculations, *Xcc*8004 spp. were cultured in nutrient broth supplemented with yeast extract (3 g of beef extract, 5 g of peptone, and 3 g of yeast extract in 1 liter of water) at 28°C. Four- to five-week-old *Arabidopsis* plants were used for bacterial growth assays. For *XVE::XopD*_*Xcc8004*_ transgenic plants, leaves were infiltrated with a bacterial suspension (2 × 10^6^ CFU mL^–1^) by using a syringe at 24 h after plants had been sprayed with 20 μM β-estradiol. After inoculation, plants were kept at 21°C in a growth chamber, and bacterial populations in leaves were determined at indicated time intervals using agar plates of nutrient broth supplemented with yeast extract containing rifampin (50 μg mL^–1^). Experiments were repeated at least 3 times.

### Yeast two-hybrid assays

AD-HFR1 and BK-XopD_*Xcc*8004_ constructs were transformed into the yeast strain AH109 by using the lithium acetate/single-stranded carrier DNA/polyethylene glycol method [34]. First, yeast cells were grown on synthetic-defined minimal yeast medium lacking leucine and tryptophan (Clontech) to maintain plasmids. Transformed colonies were further plated on synthetic-defined minimal yeast medium lacking leucine, tryptophan, and histidine (Clontech) to test the interaction between XopD_*Xcc*8004_ and HFR1.

### Subcellular localization assays

To examine the subcellular localization of HFR1-CFP and XopD_*Xcc*8004_-YFP, agroinfiltration was performed [35]. Briefly, *A*. *tumefaciens* stains carrying the *35S::HFR1-CFP* or *35S::XopD*_*Xcc8004*_ *-YFP* plasmid were inoculated into *N*. *benthamiana* leaves. Fluorescence signals were observed by confocal laser scanning microscopy at 36 h after agroinfiltration, and images were collected with the Olympus Fluoview FV1000 system.

### *In vitro* pull-down assays

GST pull-down experiments were performed by inoculating 2 μg of GST alone or GST-tagged proteins with 2 μg of MBP alone or MBP-tagged proteins in binding buffer (50 mM Tris-HCl at pH 7.5, 100 mM NaCl, 0.25% Triton X-100, 35 mM β-mercaptoethanol) for 2 h at room temperature. Next, 25 μL of glutathione-Sepharose 4B (GE Healthcare) were added, and samples were further incubated for 1 h at room temperature. After extensive wash, pulled down proteins were eluted with 2.5× sample buffer and separated on a 10% sodium dodecyl sulfate-polyacrylamide gel. Western blotting was performed using an anti-MBP antibody to detect MBP-tagged proteins (Amersham). The chemiluminescence signals generated by the ECL reagent were further examined with the ImageQuant LAS4000 mini (GE Healthcare).

### *In vitro* sumoylation assays

*In vitro* sumoylation was performed using purified recombinant proteins in a reaction buffer (50 mM Tris-HCl, pH7.4, 100 mM NaCl, 4 mM ATP, 10 mM MgCl_2_, 4 mM DTT) in a total volume of 30 μL. After incubation for 2 h at 30°C, the reaction mixtures were separated on 10% sodium dodecyl sulfate-polyacrylamide gels. MBP-HFR1 and sumoylated MBP-HFR1 were detected by western blotting using an anti-MBP antibody. The chemiluminescence signals generated by the ECL reagent were further examined with the ImageQuant LAS4000 mini (GE Healthcare).

### RNA-Seq analysis

To identify differentially expressed genes in *XVE::XopD*_*Xcc8004*_transgenic plants upon β-estradiol treatment, next-generation sequencing was done on the Hiseq 2000 (Illumina) using total RNA samples extracted with the RNeasy plant mini kit (Qiagen). For transcriptome analysis, sequence reads were aligned using CLC bio and gene expression levels were normalized as reads per kilobase of exon model per million mapped reads. Finally, the differentially expressed genes were identified by DEseq [36].

### Primers

Primer sequences for plasmid constructions and qRT-PCR analyses are listed in S1 Table.

## Results

### Expression of XopD_*Xcc*8004_ elicits a SA-mediated defense response in *Arabidopsis*

**Figure 1:**
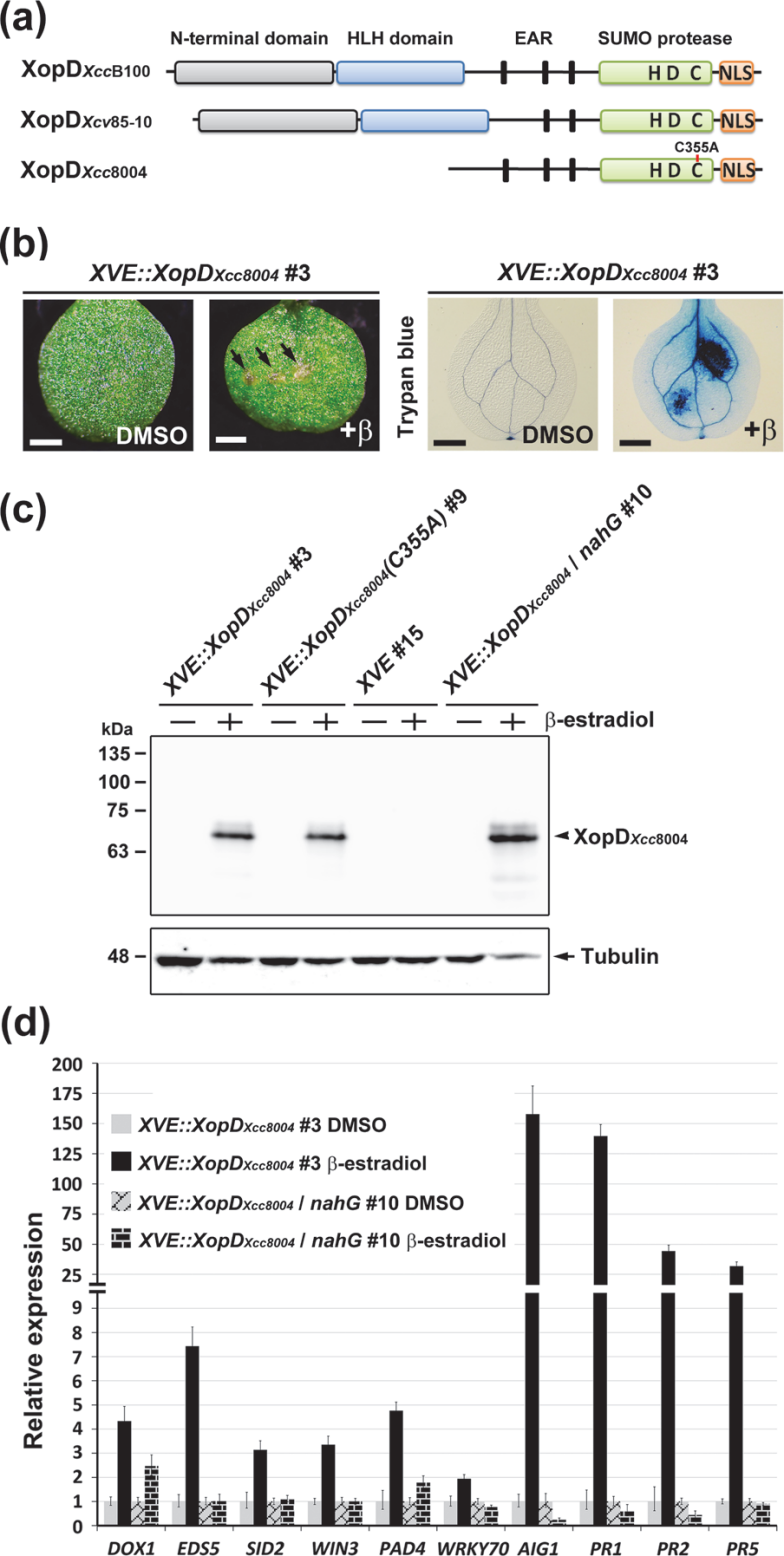
Salicylic acid-dependent defense responses were elicited by the expression of XopD_*Xcc*8004_ in *Arabidopsis*. (a) Schematic representation of XopD proteins. (b) Morphological examination and trypan blue staining of two-week-old leaves of *Arabidopsis XVE::XopD*_*Xcc8004*_ transgenic plants. Lesion-mimic phenotypes were indicated by arrows. Scale bar: 1 mm. (c) Translated products of *XopD*_*Xcc8004*_ and *XopD*_*Xcc8004*_ *(C355A)* were examined by western blotting using a specific antibody against XopD_*Xcc*8004_. Anti-tubulin was used for loading control. (d) The expression levels of genes involved in the SA-mediated defense signaling network were examined by qRT-PCR and normalized to *EF1α*. The relative expression levels of each gene in the DMSO control were set at 1.

XopD_*Xcc*8004_ from the *Xcc* strain 8004 is a shorter version of XopD, which lacks the N-terminal domain (Fig. 1A). To characterize the functions of XopD_*Xcc*8004_ in plant cells, *Arabidopsis* transgenic plants carrying an inducible *XVE::XopD*_*Xcc8004*_ transgene were generated. Here, a lesion-mimic phenotype associated with localized, necrotic spots was observed in *XVE::XopD*_*Xcc8004*_ transgenic plants after β-estradiol (inducer) treatment (Fig. 1B, C). Further examination using trypan blue staining confirmed that lesion-mimics were formed because of cell death (Figs. 1B, S1), whereas transgenic plants harboring empty vector (*XVE*) did not show cell death phenotype upon β-estradiol treatment (S2 Fig.).

To examine whether XopD_*Xcc*8004_ can trigger a SA-mediated defense response which may contribute to the hypersensitive cell death, genes involved in the SA-mediated defense-signaling network [37] were selected for quantitative reverse transcription-polymerase chain reaction (qRT-PCR) validation. Compared with dimethyl sulfoxide (DMSO) control treatment, genes involved in defense response (*AIG1* and *DOX1*), SA biosynthesis (*EDS5* and *SID2*), SA accumulation and downstream signaling (*WIN3*, *PAD4* and *WRKY70*), as well as SA-standard marker genes (*PR1*, *PR2* and *PR5*) were highly elevated in *XVE::XopD*_*Xcc8004*_ transgenic plants after β-estradiol treatment (Figs. 1D, S1). By contrast, no significant difference was observed in *XVE* (vector only) transgenic plants (S2 Fig.). Next, we transfected *XVE::XopD*_*Xcc8004*_ into an *Arabidopsis* transgenic line overexpressing bacterial salicylate hydroxylase (*nahG*). With the expression of *nahG*, the cell death phenotype and SA response-related genes induced by XopD_*Xcc*8004_ were all inhibited by *nahG* (Figs. 1D, S3). These results suggest that XopD_*Xcc*8004_ elicits a SA-dependent defense response which may contribute to the lesion-mimic phenotype observed in *XVE::XopD*_*Xcc8004*_ transgenic plants.

To investigate the genome-wide expression profile of mRNAs in response to the expression of XopD_*Xcc*8004_ in *Arabidopsis*, the total RNA from *XVE::XopD*_*Xcc8004*_ transgenic seedlings upon DMSO and β-estradiol treatments were extracted for a comparative transcriptome analysis. In summary, a total of 23.1 million reads mapped to the *Arabidopsis* genome were generated after quality trim and a total of 103 differentially expressed genes with *p* < 0.001 were identified using the DESeq method (S2 and S3 Tables). Among them, 85 genes were upregulated and 18 genes were downregulated after β-estradiol treatment. Functional annotations on the 85 up-regulated genes revealed that a total of 25 genes associated with defense responses including the SA-mediated response were highly induced by XopD_*Xcc*8004_ (Table 1).

**Table 1:** Annotation of the differentially expressed genes (*p* < 0.001) involved in plant defense responses.

Name	AGI number	Base mean		log2 Fold Change	Functional annotations
		β-estradiol	DMSO		
SBT3.3	At1g32960	2.19	434.53	7.63	Subtilase family protein
DIR5	At1g64160	2.19	354.19	7.34	Disease resistance-responsive family protein
AIG1	At1g33960	20.81	850.80	5.35	AvrRPT2-induced gene 1
PCC1	At3g22231	14.24	544.07	5.26	Pathogen and circadian controlled 1
TPS4	At1g61120	5.48	262.91	5.58	Terpene synthase 4
	At5g10760	25.20	670.96	4.74	Eukaryotic aspartyl protease family protein
GSTF3	At2g02930	16.43	406.23	4.63	Glutathione S-transferase F3
	At5g03350	39.44	746.73	4.24	Legume lectin family protein
GSTU4	At2g29460	23.00	461.91	4.33	Glutathione S-transferase tau 4
NIT2	At3g44300	261.81	3868.75	3.89	Nitrilase 2
RLP23	At2g32680	12.05	272.95	4.50	Receptor-like protein 23
ELI3	At4g37990	5.48	175.27	5.00	Elicitor-activated gene 3–2
	At1g13609	4.38	157.93	5.17	Defensin-like (DEFL) family protein
ANK	At5g54610	6.57	188.05	4.84	Ankyrin
CRK7	At4g23150	1.10	102.24	6.54	Cysteine-rich receptor-like protein kinase 7
PR2	At3g57260	47.10	709.30	3.91	Beta-1,3-glucanase 2
FMO1	At1g19250	18.62	340.50	4.19	Flavin-dependent monooxygenase 1
CRK4	At3g45860	8.76	189.88	4.44	Cysteine-rich receptor-like protein kinase 4
CYP71A13	At2g30770	15.34	262.91	4.10	Cytochrome P450, family 71, subfamily A, polypeptide 13
WAKL10	At1g79680	8.76	179.84	4.36	Wall-associated kinase-like 10
PDF1.4	At1g19610	27.39	355.11	3.70	Arabidopsis defensin-like protein
PNP-A	At2g18660	12.05	191.70	3.99	Plant natriuretic peptide A
RLP38	At3g23120	2.19	85.81	5.29	Receptor-like protein 38
	At5g24200	4.38	109.54	4.64	Alpha/beta-hydrolases superfamily protein
	At3g04220	9.86	163.40	4.05	Disease resistance protein (TIR-NBS-LRR) family

AGI, *Arabidopsis* Genome Initiative; Base mean, the number of reads divided by the size factor (normalization constant) of sample; Fold change, β-estradiol base mean/DMSO base mean.

### Suppression of *Xcc*8004 growth by XopD_*Xcc*8004_

**Figure 2:**
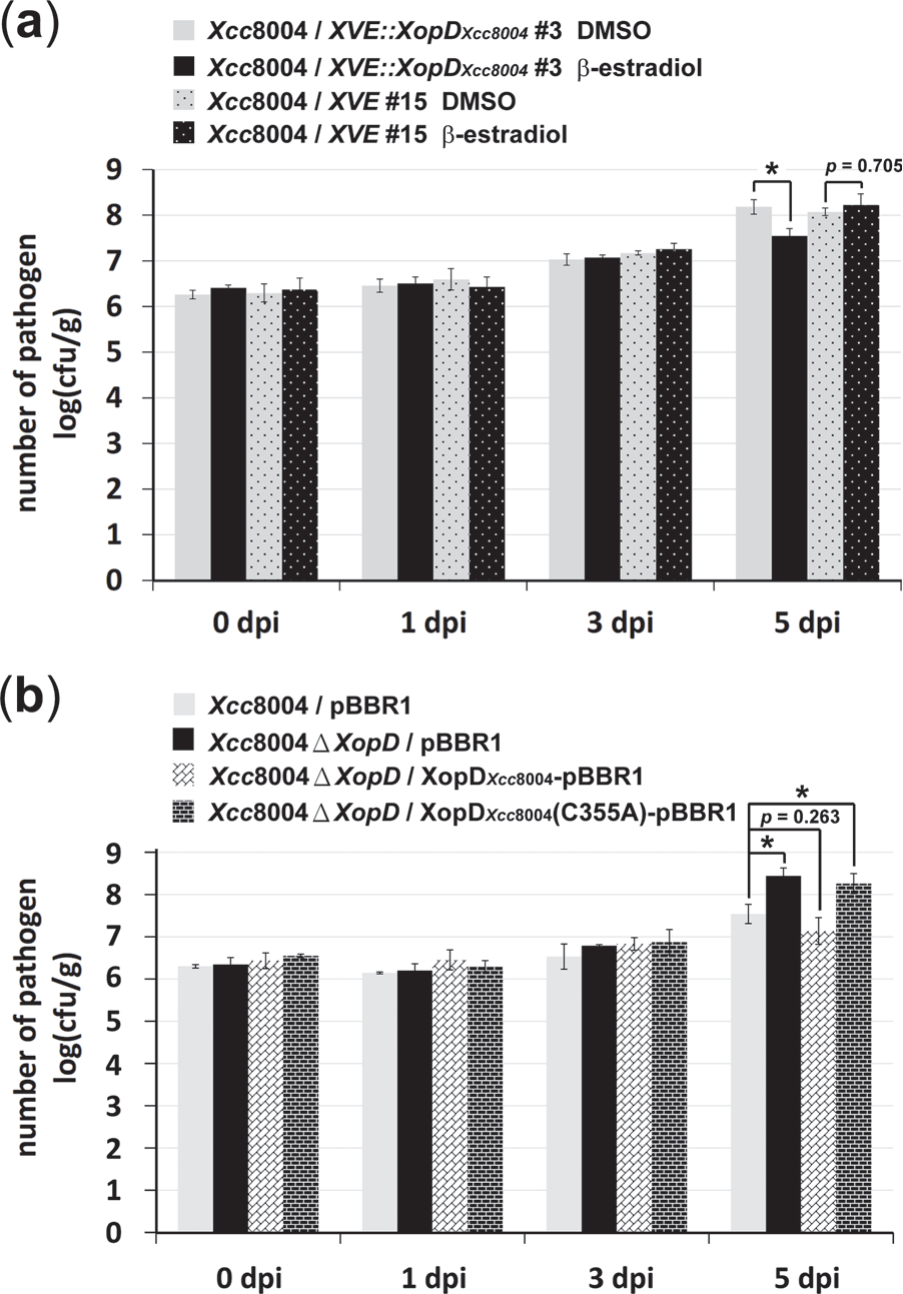
XopD_*Xcc*8004_ suppresses the virulence of the *Xcc*8004. (a) Bacterial growth of the *Xcc*8004 strain in *Arabidopsis* transgenic plants. DMSO or β-estradiol was applied 24 h before bacterial inoculation. Hand-infiltrated leaves were collected at the indicated times for measuring the *in planta* growth of bacterial populations. (b) Bacterial growth of *Xcc*8004 spp. in *Arabidopsis* plants were measured to examine the effects of XopD_*Xcc*8004_ on the virulence of *Xcc*8004. Statistically significant differences were determined using one-way ANOVA (* indicates *p* < 0.05).

Because the lesion-mimic phenotype accompanied by the up-regulation of SA response-related genes in *XopD*_*Xcc8004*_-transgenic plants resembles the hypersensitive response (HR) in pathogen infection, we hypothesize that the expression of XopD_*Xcc*8004_ in *Arabidopsis* may result in enhanced resistance against bacterial pathogens. In order to examine the effect of XopD_*Xcc*8004_ on the resistance of *Arabidopsis*, multiplication of the *Xcc*8004 strain on *XVE::XopD*_*Xcc8004*_ transgenic plants was tested after β-estradiol treatment. Compared with *XVE* transgenic plants, multiplication of the *Xcc*8004 strain was suppressed by the expression of XopD_*Xcc*8004_ *in planta* at 5 days post inoculation (dpi) (Fig. 2A).

We further generated a *xopD* null mutant by homologous recombination to validate the function of XopD_*Xcc*8004_ in suppressing the virulence of *Xcc*8004. Here, *Arabidopsis* WT leaves inoculated with *Xcc*8004 *ΔXopD* strain exhibited a higher titer of bacteria at 5 dpi than those inoculated with the *Xcc*8004 strain (Fig. 2B). This phenotype was able to be complemented when *Xcc*8004 *ΔXopD* strain was transformed with a broad host plasmid (pBBR1) expressing XopD_*Xcc*8004_ (Fig. 2B). Taken together, these results suggest that XopD_*Xcc*8004_ acts as a negative factor in suppressing the growth of *Xcc*8004.

### XopD_*Xcc*8004_-eliciting defense responses are mainly dependent on the SUMO protease activity

**Figure 3:**
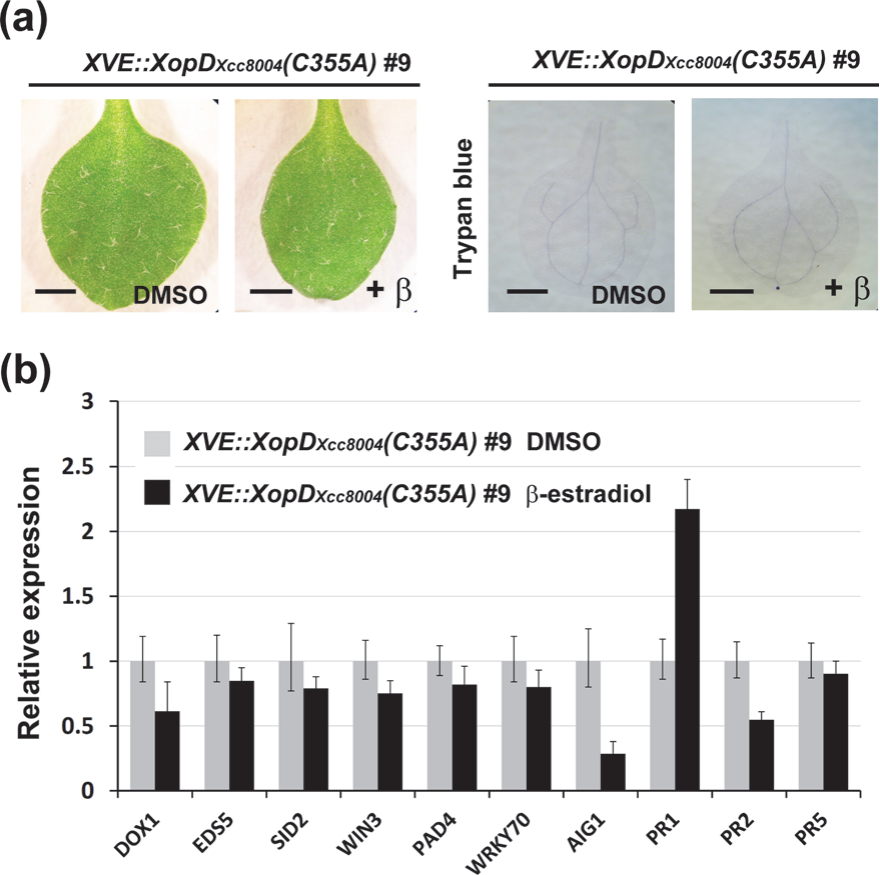
XopD_*Xcc*8004_(C355A) loses the activity for activating plant immunity. (a) Morphological examination and trypan blue staining of two-week-old leaves of *Arabidopsis XVE::XopD*_*Xcc8004*_ *(C355A)* transgenic plants. Scale bar: 1 mm. (b) The expression levels of genes involved in the SA-mediated defense signaling network were examined by qRT-PCR and normalized to *EF1α*. The relative expression levels of each gene in the DMSO control were set at 1.

To examine whether the SUMO protease activity of XopD_*Xcc*8004_ is required for eliciting the plant immunity, *XVE::XopD*_*Xcc8004*_ *(C355A)* transgenic plants were generated, in which mutation of the conserved cysteine (C355A) in XopD_*Xcc*8004_ was not able to hydrolyse the SUMO substrates. Compared with *XVE::XopD*_*Xcc8004*_ transgenic plants, no hypersensitive cell death was observed in *XVE::XopD*_*Xcc8004*_ *(C355A)* transgenic plants after β-estradiol treatment (Figs. 1B, 3A, S4). Further investigation of the gene expression involved in defense responses revealed that the C355A mutation largely suppressed the ability of XopD_*Xcc*8004_ in eliciting the expression of SA response-related genes in *Arabidopsis* (Figs. 3B, S4). Next, we transformed *Xcc*8004 *ΔXopD* strain with a broad host plasmid to express XopD_*Xcc*8004_(C355A). However, *Arabidopsis* WT leaves inoculated with *Xcc*8004 *ΔXopD*/XopD_*Xcc*8004_(C355A) strain still exhibited a higher titer of bacteria at 5 dpi as well as those inoculated with *Xcc*8004 *ΔXopD* strain (Fig. 2B). These results suggest that the SUMO protease activity of XopD_*Xcc*8004_ is required for eliciting a SA-mediated defense response in *Arabidopsis* and suppressing the virulence of *Xcc*8004.

### XopD_*Xcc*8004_ interacts with *Arabidopsis* HFR1

**Figure 4:**
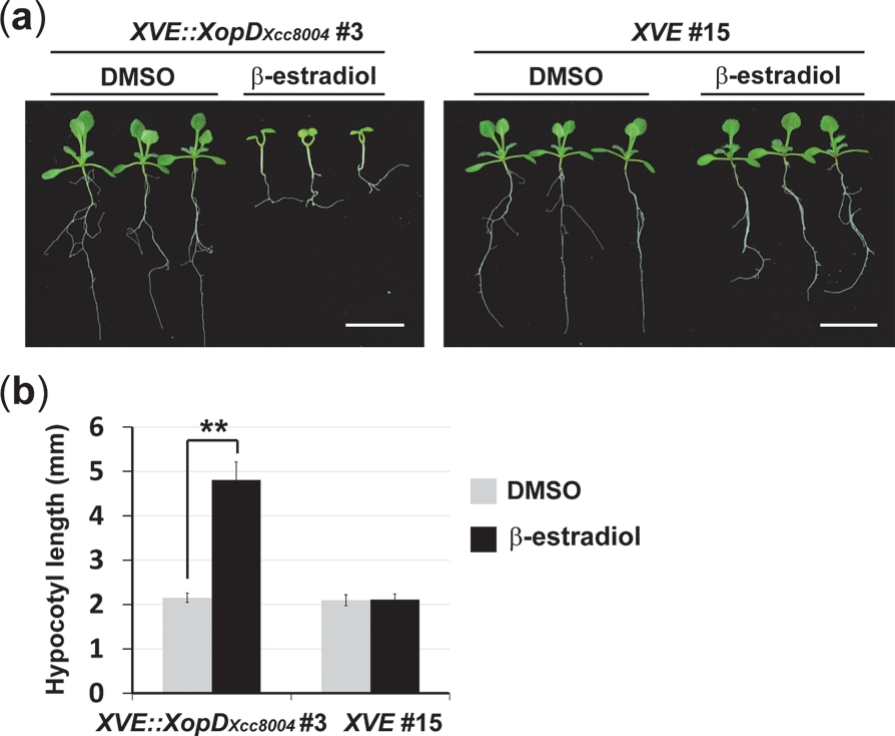
Expression of XopD_*Xcc*8004_ induces a long hypocotyl phenotype in *Arabidopsis*. (a) Phenotypes of 12-day-old *Arabidopsis* transgenic plants carrying a *XopD*_*Xcc8004*_ gene driven by the inducible *XVE* promoter. Scale bars: 8 mm. (b) Average hypocotyl lengths of seedlings grown on medium containing DMSO or 20 μM β-estradiol. Statistically significant differences were determined using one-way ANOVA (** indicates *p* < 0.005).

**Figure 5:**
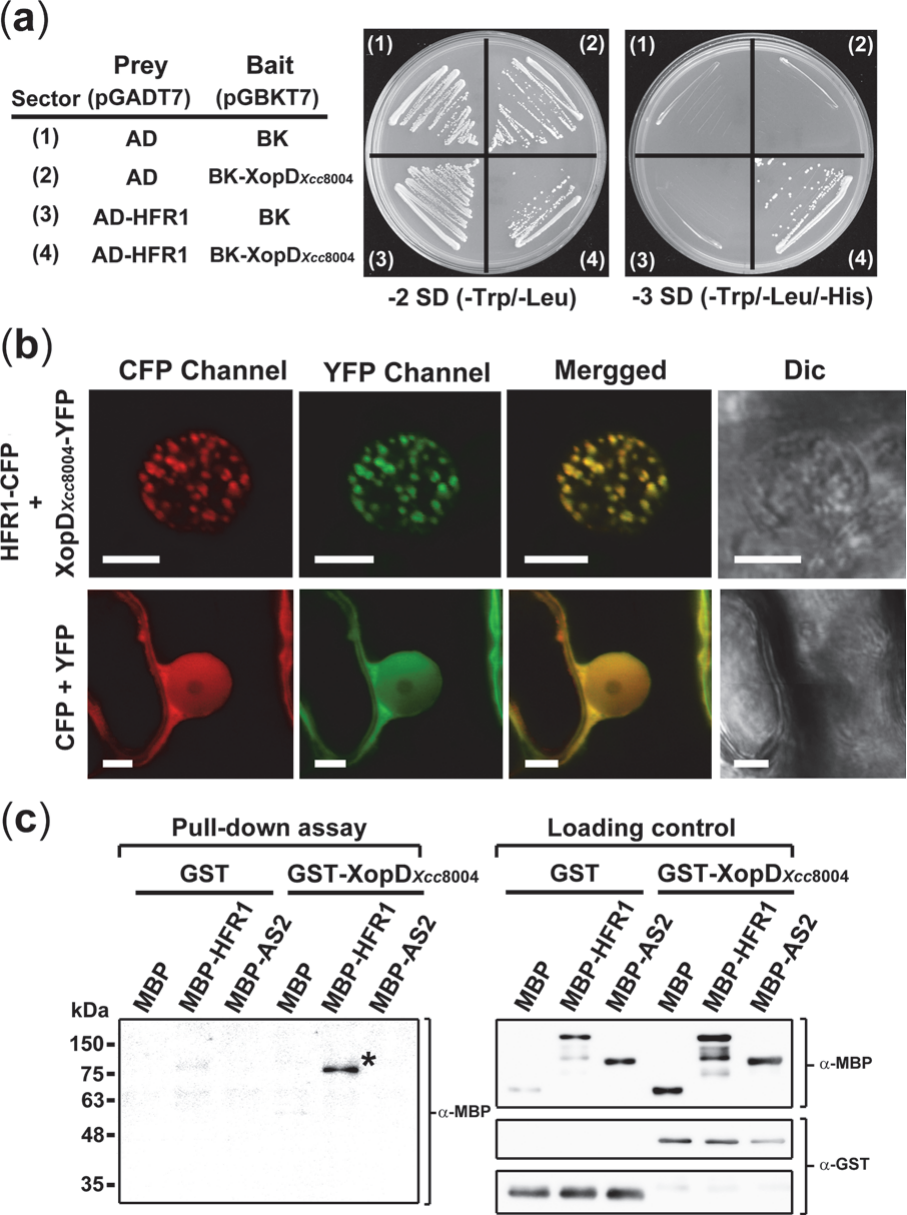
XopD_*Xcc*8004_ interacts with HFR1. (a) Investigation of the interaction between HFR1 and XopD_*Xcc*8004_ by yeast two-hybrid analysis. Yeast strains transformed with two plasmids (prey and bait) were plated onto synthetic-defined (SD) minimal medium lacking tryptophan/leucine or tryptophan/leucine/histidine. (b) *Nicotiana benthamiana* leaves were co-infiltrated with agrobacterium carrying *35S::XopD*_*Xcc8004*_ *-YFP* and *35S::HFR1-CFP*. Fluorescence and differential interference contrast (DIC) images were obtained by confocal laser scanning microscopy. *35S::YFP* and *35S::CFP* were used for control. Scale bars: 15 μm. (c) Investigation of the interaction between HFR1 and XopD_*Xcc*8004_ by using pull-down assays. Briefly, 2 μg of GST or GST fusion proteins were used to pull down 2 μg of MBP or MBP fusion proteins, and an anti-MBP antibody was used to detect the associated proteins by western blotting (the left panel). The right panels present the input proteins examined by anti-MBP or anti-GST antibodies. The asterisk indicates the protein signal of MBP-HFR1.

Unexpectedly, a long hypocotyl phenotype associated with the lesion-mimic phenotype was observed with the expression of XopD_*Xcc*8004_ in *Arabidopsis* (Fig. 4A). Compared with *XVE* transgenic plants, the hypocotyl length of *XVE::XopD*_*Xcc8004*_ seedlings reached to 4.5 mm at 14 days after germination in medium containing β-estradiol (Figs. 4B, S5). We noted that the long hypocotyl phenotype elicited by the expression of XopD_*Xcc*8004_ similar to those observed in *siz1–2* carrying a partially functional *SIZ1* ^*phd*^ [38]. SIZ1^phd^ contains a C134Y mutation in the zinc finger motif required for the SUMO E3 ligase activity of SIZ1. Cheong et al. found that expression of *SIZ1* ^*phd*^ in the *siz1–2* mutant created a light-dependent long hypocotyl phenotype. Therefore, we investigated whether light signaling components were potential substrates of XopD_*Xcc*8004_. Here, several key components involved in the light signaling pathway including HY5, LAF1, Fin219, PAT3, SPA1, and HFR1were analyzed for their interaction with XopD_*Xcc*8004_ using the yeast two-hybrid assay. Among them, HFR1, a basic helix-loop-helix transcription factor, showed a positive interaction with XopD_*Xcc*8004_ in selection medium lacking tryptophan/leucine/histidine (Fig. 5A).

Next, we investigated whether XopD_*Xcc*8004_ can colocalize with HFR1 in plant cells. HFR1 has been shown to localize to nucleus in subnuclear foci [27], whereas XopD_*Xcc*8004_ was localized to the nucleus in a homogeneous pattern [17]. If XopD_*Xcc*8004_ can interact with HFR1, the coexpression of HFR1 may cause the relocalization of XopD_*Xcc*8004_ to HFR1-containing nuclear foci. Here, we coexpressed XopD_*Xcc*8004_-yellow fluorescence protein (YFP) and HFR1-cyan fluorescent protein (CFP) in *Nicotiana benthamiana* cells using agroinfiltration and found that XopD_*Xcc*8004_-YFP was colocalized with HFR1-CFP in nuclear foci (Fig. 5B). To further examine whether XopD_*Xcc*8004_ can directly interact with HFR1 *in vitro*, a pull-down assay was performed with purified recombinant proteins. Fig. 5C shows that maltose-binding protein (MBP)-HFR1 was specifically pulled down by glutathione S-transferase (GST)-XopD_*Xcc*8004_ but not by GST alone. By contrast, no signal was observed when the negative control proteins MBP and MBP-AS2 were used in the assay (Fig. 5C).

To examine whether the long hypocotyl phenotype observed in *XVE::XopD*_*Xcc8004*_ seedlings is dependent on the SUMO protease activity of XopD_*Xcc*8004_, we investigated the phenotype of *XVE::XopD*_*Xcc8004*_ *(C355A)* transgenic plants. However, a long hypocotyl phenotype was also observed in *XVE::XopD*_*Xcc8004*_ *(C355A)* seedlings (S5 Fig.). This result suggests that the long hypocotyl phenotype caused by XopD _Xcc8004_ is not simply due to the SUMO protease activity.

### K72 in HFR1 is desumoylated by XopD_*Xcc*8004_ *in vitro*

**Figure 6:**
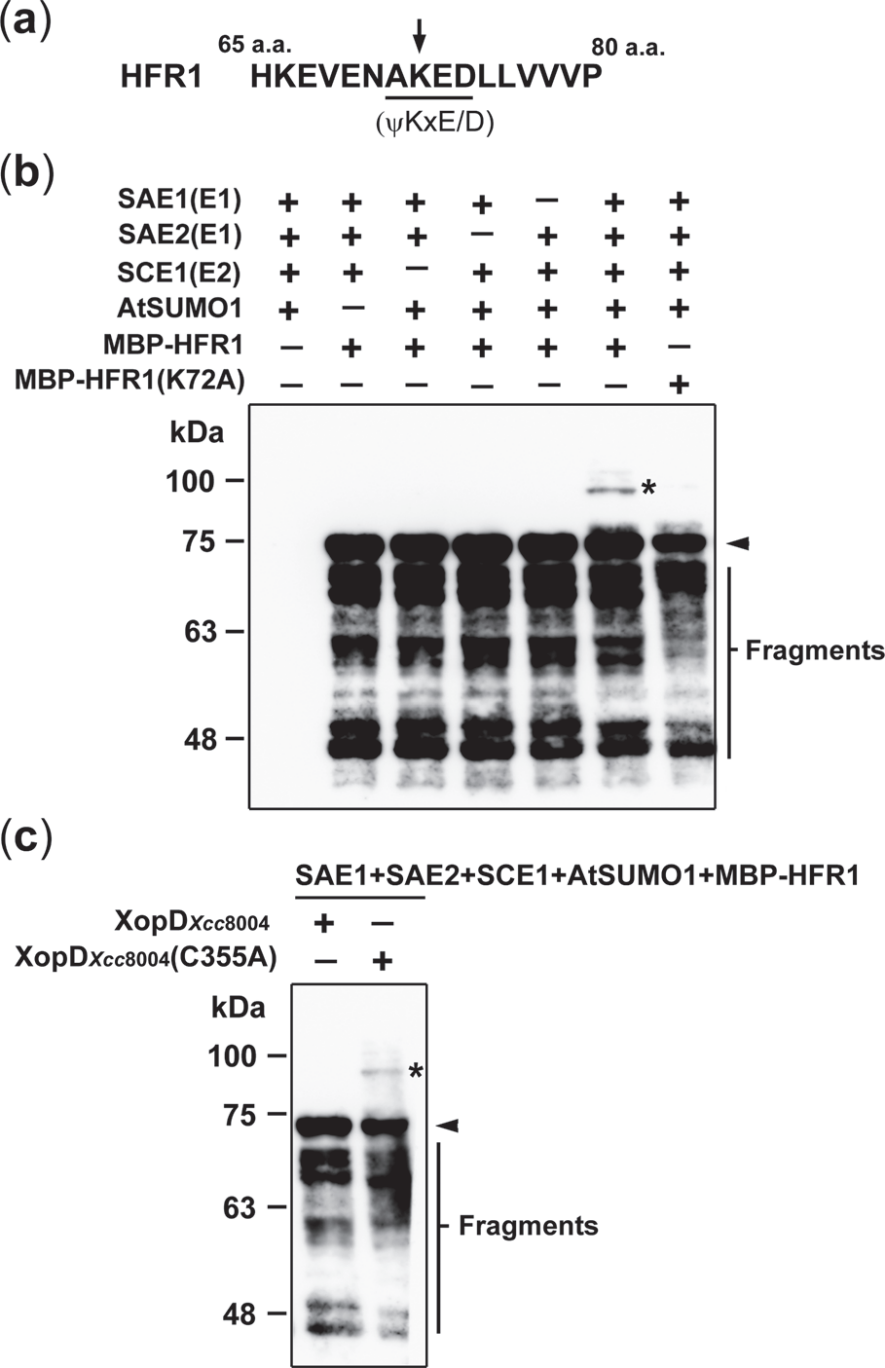
K72 in HFR1 is desumoylated by XopD_*Xcc*8004_ *in vitro*. (a) The deduced amino acid sequence of HFR1 from 65 to 80 a.a. The arrow indicates a putative sumoylation site at K72 of the consensus motif ΨKxE/D of HFR1. (b) An *in vitro* sumoylation system was established by using purified recombinant proteins, including *Arabidopsis* SAE1, SAE2, SCE1, and AtSUMO1. MBP-HFR1 or MBP-HFR1(K72A) were used as potential substrates for sumoylation and detected with an anti-MBP antibody. (c) Together with SAE1, SAE2, SCE1, AtSUMO1, and MBP-HFR1, XopD_*Xcc*8004_ was added to the reaction mixture to investigate the sumoylation of HFR1. XopD_*Xcc*8004_(C355A), a SUMO protease mutant, was used as control. Asterisks indicate sumoylated MBP-HFR1 proteins. Arrowheads indicate unmodified MBP-HFR1 proteins. Signals below the unmodified MBP-HFR1 proteins were degraded products of purified MBP-HFR1 proteins.

Because XopD_*Xcc*8004_ has been shown to possess SUMO protease activity [16], [17], the interaction between XopD_*Xcc*8004_ and HFR1 prompted us to examine whether HFR1 can be modified with SUMO and further desumoylated by XopD_*Xcc*8004_. Here, the examination of the deduced amino acid sequence of HFR1 revealed a probable sumoylation site at lysine 72 (K72) in the consensus motif ΨKxE/D (where Ψ is a large and hydrophobic amino acid and x is any amino acid) (Fig. 6A). To address whether HFR1can be modified with SUMO, an *in vitro* sumoylation assay was performed with *Arabidopsis* SAE1/SAE2 (SUMO-activating E1), SCE1 (SUMO-conjugating E2), AtSUMO1, and MBP-HFR1. Fig. 6B shows that a clear mobility shift of MBP-HFR1was detected after incubation with the components of the *Arabidopsis* sumoylation cascade, and the shift was in a molecular mass consistent with mono-SUMO modification. Compared with MBP-HFR1, no mobility shift was detected for MBP-HFR1(K72A) in the *in vitro* sumoylation system (Fig. 6B). These results indicate that the K72 of HFR1 is the principal site for SUMO conjugation. Next, we examined whether the sumoylated HFR1 can be desumoylated by XopD_*Xcc*8004_. Fig. 6C shows that no sumoylated MBP-HFR1 was detected when XopD_*Xcc*8004_, but not the catalytic mutant XopD_*Xcc*8004_(C355A), was present in the *in vitro* sumoylation reaction. These results indicate that XopD_*Xcc*8004_ catalyzes the SUMO hydrolysis from the K72 of HFR1.

### *hfr1–201* increases resistance to *Xcc*8004

**Figure 7:**
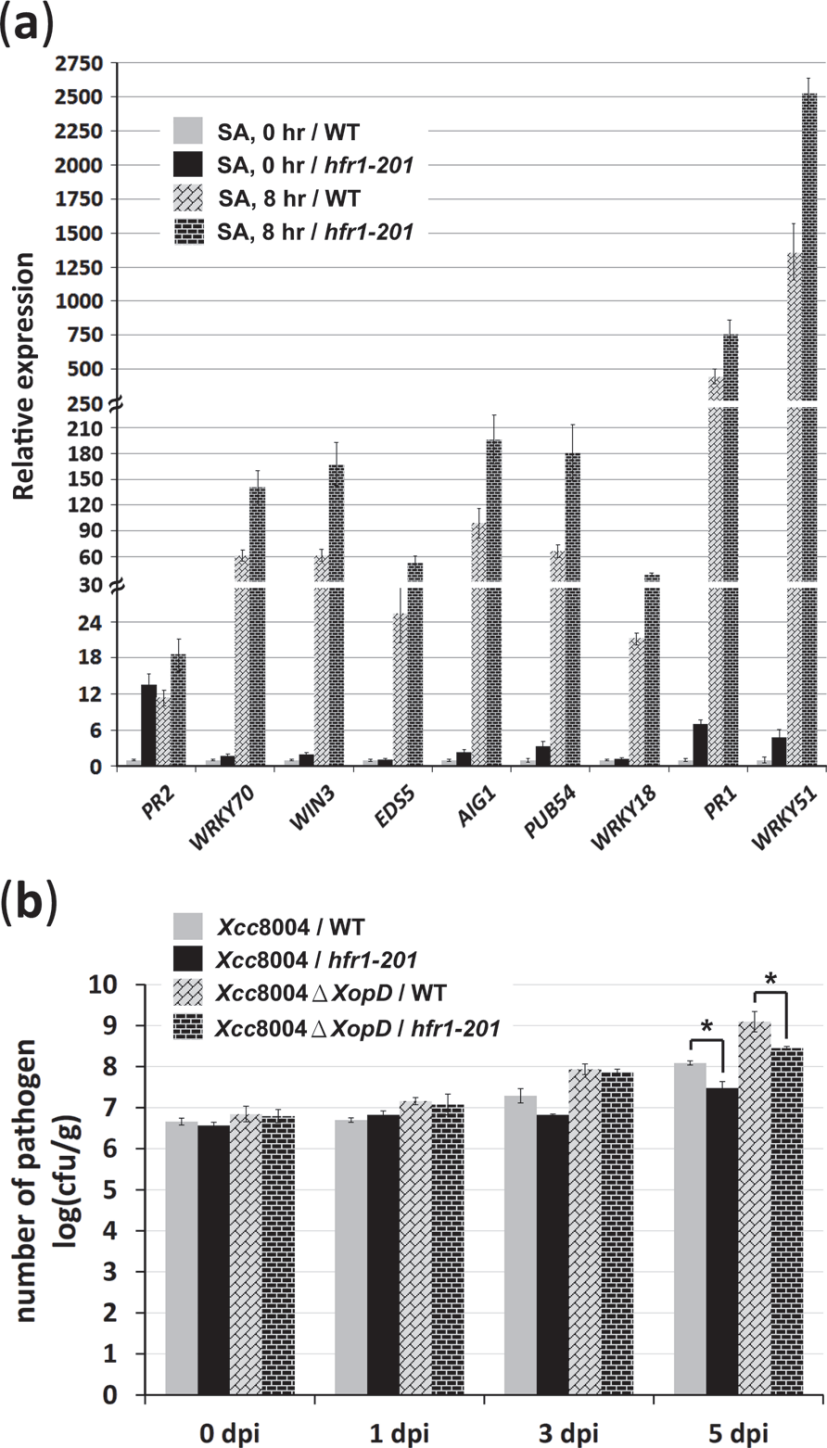
*hfr1–201* increases plant immunity against *Xcc*8004 spp. (a) *Arabidopsis* WT and *hfr1–201* mutant plants treated with (8 hr) or without (0 hr) 2 mM SA were collected for total RNA extraction. The expression levels of genes involved in the SA-mediated defense signaling network were examined by qRT-PCR and normalized to *EF1α*. The relative expression levels of each gene in the WT plants without SA treatment were set at 1. (b) Bacterial growth in *Arabidopsis* WT and *hfr1–201* mutant plants were measured to examine the effects of HFR1 on the resistance of *Arabidopsis* against *Xcc*8004 spp. Hand-infiltrated leaves were collected at the indicated times for measuring the *in planta* growth of bacterial populations. Statistically significant differences were determined using one-way ANOVA (* indicates *p* < 0.05).

Based on the interaction between HFR1and XopD_*Xcc*8004_, we propose that HFR1 may play a role in the plant immune response. To this end, we monitored the expression levels of genes involved in the SA-mediated defense-signaling network in *hfr1–201* mutants and WT plants. Irrespective of treatment with or without 2 mM SA, we found that the levels of *PR2*, *WRKY70*, *WIN3*, *EDS5*, *AIG1*, *PUB54*, *WRKY18*, *PR1*, and *WRKY51* transcripts are higher in the *hfr1–201* mutant than in WT plants (Fig. 7A). We further investigated the growth of *Xcc*8004 WT and *ΔXopD* mutant strains in the *hfr1–201* mutant. Compared with WT plants, multiplication of *Xcc*8004 and *Xcc*8004 *ΔXopD* strains was suppressed in the *hfr1–201* mutant (Fig. 7B). These results suggest that HFR1 is required for modulating the defense response in *Arabidopsis*, and the loss-of-function mutant in the *HFR1* increases resistance to *Xcc*8004 spp.

## Discussion

XopD is composed of an N-terminal domain, ERF-associated amphiphilic repression motifs, and a C-terminal SUMO protease domain. In XopD_*Xcv*85–10_ and XopD_*Xcc*B100_, the N-terminal domain has been suggested to play a role in specifying substrate recognition and modulating SUMO protease activity [16], [18]. However, XopD_*Xcc*8004_ was considered as a shorter version of XopD that does not contain an N-terminal domain [17], [19]. Using a transgenic approach, we found that possessing a C-terminal SUMO protease domain, the XopD_*Xcc*8004_-overexpressing transgenic plants showed morphological and molecular phenotypes similar to those of the mutants defective in the sumoylation machinery. In *Arabidopsis*, mutants defective in the sumoylation machinery, e.g., *siz1* and *sum1sum2* mutants, display localized programmed cell death and elevated accumulation of SA, which contribute to the enhanced resistance against bacterial attacks [6], [7]. In this study, we showed that the expression of XopD_*Xcc*8004_ in *Arabidopsis* elicited a lesion-mimic phenotype associated with increased expression of disease-response genes (Fig. 1B). Moreover, the multiplication of the *Xcc*8004 strain was suppressed in XopD_*Xcc*8004_-overexpressing transgenic plants (Fig. 2A), and *Xcc*8004 *ΔXopD* displayed a higher bacterial growth rate than *Xcc*8004 (Fig. 2B). Different from XopD_*Xcv*_ and XopD_*Xcc*B100_, which contain a complete N-terminal domain and are successful in suppressing anti-*Xcv* and anti-*Xcc*B100 immunities [16], [18], XopD_*Xcc*8004_ induced host defense responses to inhibit *Xcc*8004 growth. Although it is still unknown how the N-terminal domain modulates the SUMO protease activity of XopD, it is clear that impairment in the N-terminal domain of XopD not only loses the ability to suppress host immunity, but also elicits host defense responses.

Based on the results that the catalytic mutant XopD_*Xcc*8004_(C355A) was not able to elicit HR and plant immunity (Fig. 3A, B), we suggest that the activity of XopD_*Xcc*8004_ in eliciting host defense responses is mainly dependent on the SUMO protease activity. Yet, the substrates of XopD_*Xcc*8004_ functioning in plant defense responses remain largely unknown. Nevertheless, we identified that HFR1 was a potential nuclear substrate of XopD_*Xcc*8004_ and could be modified by sumoylation (Figs. 5, 6). With the *in vitro* assay, we found that the sumoylated-residue K72 within the consensus sumoylation motif of HFR1 was desumoylated by XopD_*Xcc*8004_ through the SUMO protease activity (Fig. 6B, C). Although the direct impact of SUMO protease activity of XopD_*Xcc*8004_ on the function of HFR1 remains unknown, we observed that the loss-of-function mutation in *HFR1* gene accelerated SA-mediated responses and increased the resistance against *Xcc*8004 (Fig. 7A, B). These results clearly indicate that HFR1 plays a role in repression of defense responses in plants.

HFR1 is an important regulator involved in photomorphogenesis and regulated by ubiquitin-mediated degradation through the coat protein complex I [27], [39], [40]. Because the highly unstable character of HFR1 [41], it is a technical challenge to provide a tight connection between XopD_*Xcc*8004_ and HFR1 during infection. Thus, further experiments are required to demonstrate the function of sumoylation on the manipulation of the activity of HFR1. Sumoylation has been shown to play a role in mediating the transcriptional repression activity of many transcription factors [42–46]. Therefore, we do not exclude the possibility that the repression activity of HFR1 on defense responses requires a modification of SUMO. In fact, our data only explain a possible mechanism for XopD_*Xcc*8004_-triggered host defense responses, and we do not exclude the possibility that XopD_*Xcc*8004_ may have several targets in plants. Thus, we still observed a higher growth rate of *Xcc*8004 even in the *hfr1–201* mutant (Fig. 7B).

Recently, Tan et al. (2014) reported that XopD_*Xcc*8004_ triggers plant disease tolerance by targeting DELLA proteins [47]. In their study, they did not observe the significant difference in bacterial titers between *Xcc*8004 and *Xcc*8004 *ΔXopD*. This difference may be depending on the different experimental conditions used in our study. Here, a syringe infiltration method and a higher titer of bacterial suspension (2 × 10^6^ CFU mL^–1^) were used. Nevertheless, our findings are not in conflict with the fact that XopD_*Xcc*8004_ may act as a virulence-controlling factor by interfering with plant defense responses.

Recent studies on plant-pathogen interaction have provided new insights into fundamental cellular processes in plants [48], [49]. In this study, we identified a dual role of HFR1 in development and immunity. This finding indicates that HFR1 is required for the fine-tuning of the immune response, as well as contributes to our knowledge of the crosstalk between the light-signaling pathway and immune response.

## Supporting Information

S1 Fig Salicylic acid-dependent defense responses were elicited by the expression of XopD_*Xcc*8004_ in *Arabidopsis*. (a) Trypan blue staining of two-week-old leaves of *Arabidopsis XVE::XopD*_*Xcc8004*_ transgenic plants. Scale bar: 1 mm. (b) Translated products of *XopD*_*Xcc8004*_ were examined by western blotting using a specific antibody against XopD_*Xcc*8004_ and indicated by an arrow. Rubisco large subunit (RBCL) stained with coomassie brilliant blue served as a loading control. (c) The expression levels of genes involved in the SA-mediated defense signaling network were examined by qRT-PCR and normalized to *EF1α*. The relative expression levels of each gene in the DMSO control were set at 1.
(TIF)

S2 Fig Transgenic plants harboring empty vector (*XVE*) did not show cell death phenotype upon β-estradiol treatment and elicited defense responses. (a) Trypan blue staining of two-week-old leaves of *Arabidopsis XVE* transgenic plants. Scale bar: 1 mm. (b) Expression levels of genes involved in the SA-mediated defense signaling network were examined by qRT-PCR and normalized to *EF1α*. The relative expression levels of each gene in the DMSO control were set at 1.
(TIF)

S3 Fig Salicylic acid-dependent defense responses elicited by the expression of XopD_*Xcc*8004_ were inhibited by *nahG*. (a) Trypan blue staining of two-week-old leaves of *Arabidopsis XVE::XopD*_*Xcc8004*_ / *nahG* transgenic plants. Scale bar: 1 mm. (b) Translated products of *XopD*_*Xcc8004*_ were examined by western blotting using a specific antibody against XopD_*Xcc*8004_ and indicated by an arrow. Rubisco large subunit (RBCL) stained with coomassie brilliant blue served as a loading control. (c) The expression levels of genes involved in the SA-mediated defense signaling network were examined by qRT-PCR and normalized to *EF1α*. The relative expression levels of each gene in the DMSO control were set at 1.
(TIF)

S4 Fig XopD_*Xcc*8004_(C355A) loses the activity for activating plant immunity. (a) Morphological examination and trypan blue staining of two-week-old leaves of *Arabidopsis XVE::XopD*_*Xcc8004*_ *(C355A)* transgenic plants. Scale bar: 1 mm. (b) Translated products of *XopD*_*Xcc8004*_ *(C355A)* were examined by western blotting using a specific antibody against XopD_*Xcc*8004_ and indicated by an arrow. Rubisco large subunit (RBCL) stained with coomassie brilliant blue served as a loading control. (c) The expression levels of genes involved in the SA-mediated defense signaling network were examined by qRT-PCR and normalized to *EF1α*. The relative expression levels of each gene in the DMSO control were set at 1.
(TIF)

S5 Fig Expression of XopD_*Xcc*8004_ induces a long hypocotyl phenotype in *Arabidopsis*. (a, b) Average hypocotyl lengths of seedlings grown on medium containing DMSO (grey bars) and 20 μM β-estradiol (black bars). Statistically significant differences were determined using one-way ANOVA (** indicates *p* < 0.005).
(TIF)

S1 Table Primer sequences for plasmid constructions and qRT-PCR. (XLS)

S2 Table Reads counting (mapped read sequence statistic) for Illumina sequencing data. (XLS)

S3 Table Differentially expressed genes with *p* < 0.001. Identification genes that were differentially expressed in *Arabidopsis* after XopD_*Xcc*8004_ were induced by β-estradiol.
(XLS)
