# Supplementary figures and images for: Arabidopsis HFR1 Is a Potential Nuclear Substrate Regulated by the Xanthomonas Type III Effector XopDXcc8004
Source: PLoS One. 2015 Feb 3;10(2):e0117067. doi: 10.1371/journal.pone.0117067 (PMC4315394; doi:10.1371/journal.pone.0117067)

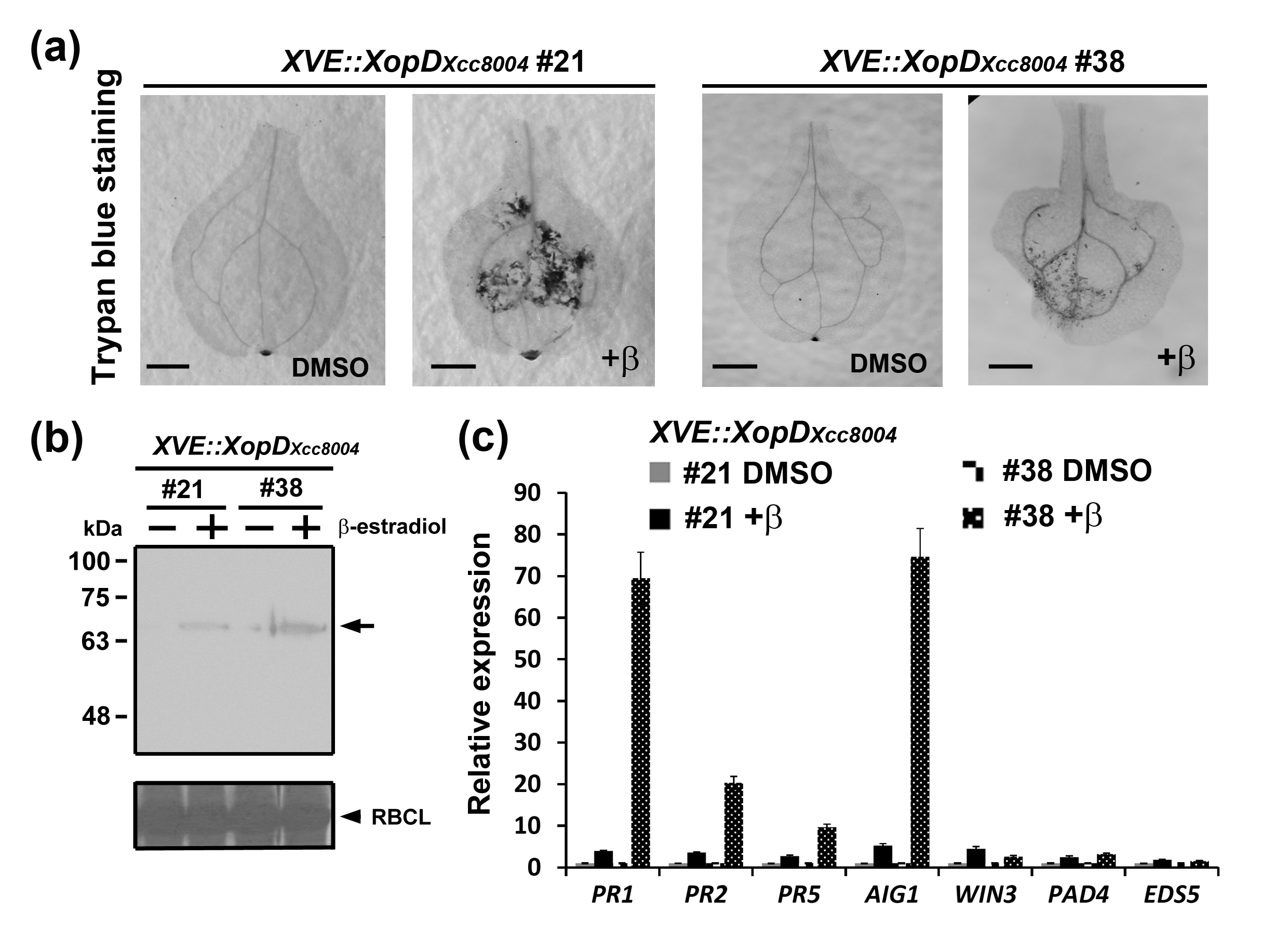

Supplement: S1 Fig — (a) Trypan blue staining of two-week-old leaves of Arabidopsis XVE::XopD Xcc8004 transgenic plants. Scale bar: 1 mm. (b) Translated products of XopD Xcc8004 were examined by western blotting using a specific antibody against XopDXcc8004 and indicated by an arrow. Rubisco large subunit (RBCL) stained with coomassie brilliant blue served as a loading control. (c) The expression levels of genes involved in the SA-mediated defense signaling network were examined by qRT-PCR and normalized to EF1α. The relative expression levels of each gene in the DMSO control were set at 1. (TIF) [file pone.0117067.s001.tif]

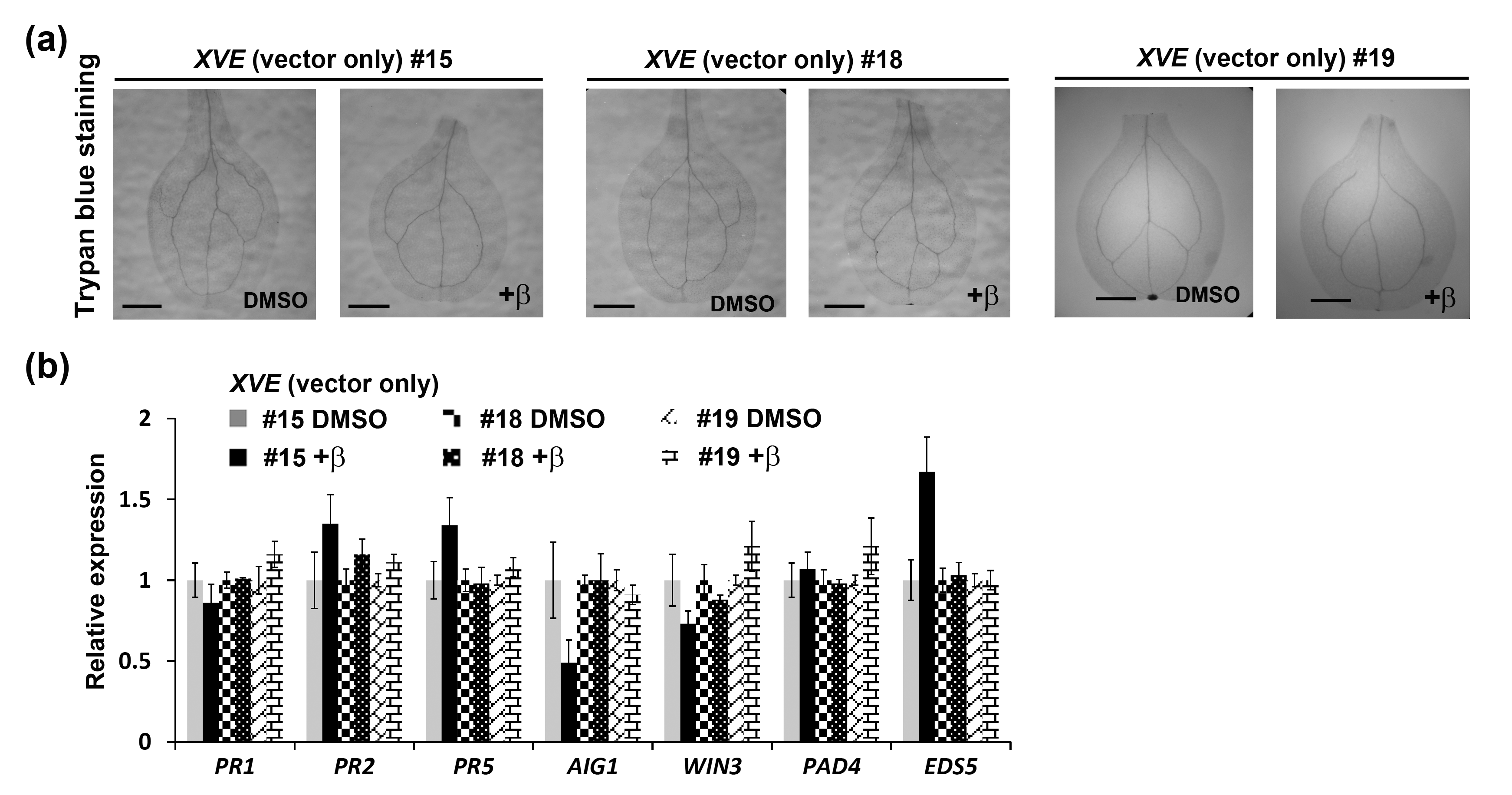

Supplement: S2 Fig — (a) Trypan blue staining of two-week-old leaves of Arabidopsis XVE transgenic plants. Scale bar: 1 mm. (b) Expression levels of genes involved in the SA-mediated defense signaling network were examined by qRT-PCR and normalized to EF1α. The relative expression levels of each gene in the DMSO control were set at 1. (TIF) [file pone.0117067.s002.tif]

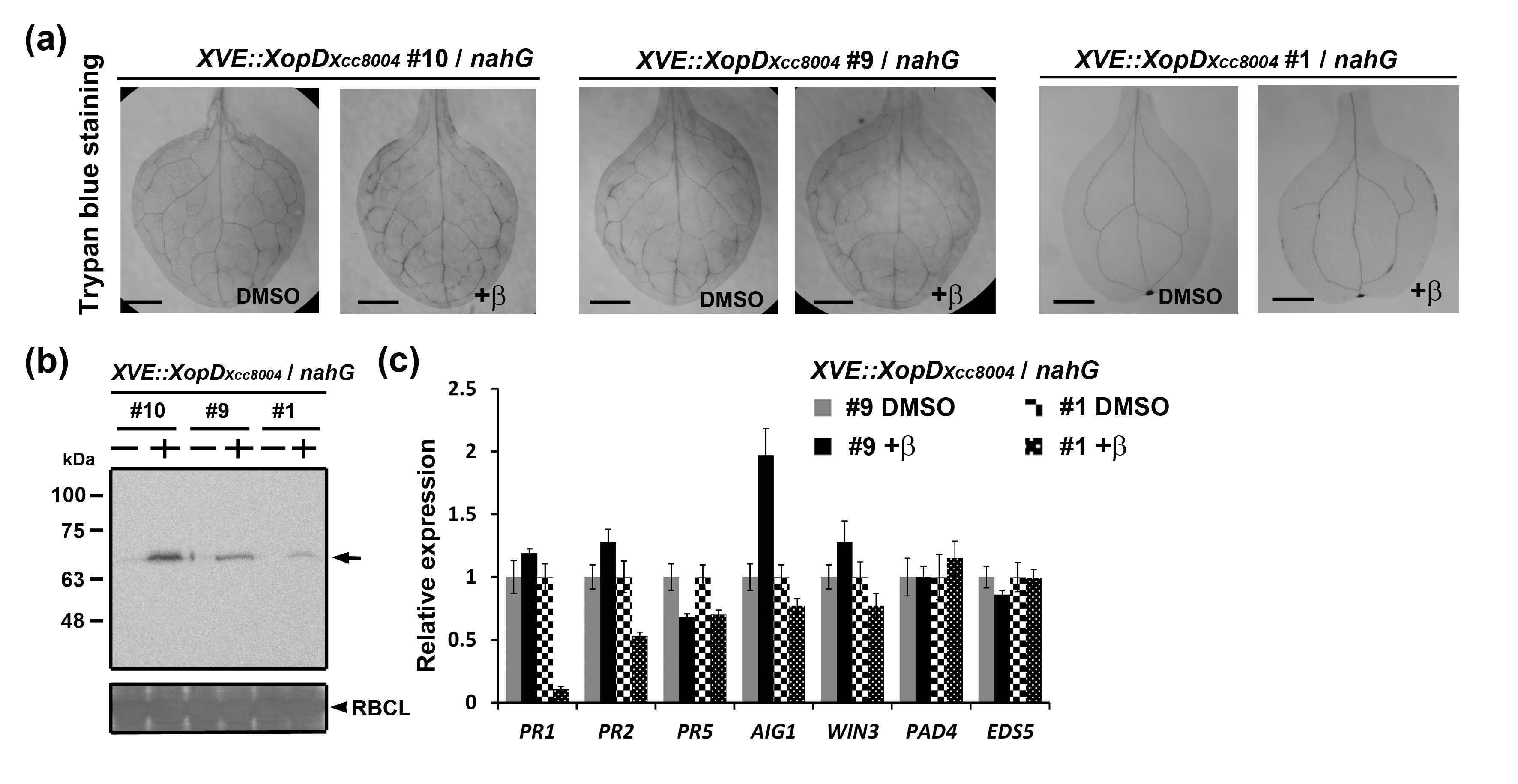

Supplement: S3 Fig — (a) Trypan blue staining of two-week-old leaves of Arabidopsis XVE::XopD Xcc8004 / nahG transgenic plants. Scale bar: 1 mm. (b) Translated products of XopD Xcc8004 were examined by western blotting using a specific antibody against XopDXcc8004 and indicated by an arrow. Rubisco large subunit (RBCL) stained with coomassie brilliant blue served as a loading control. (c) The expression levels of genes involved in the SA-mediated defense signaling network were examined by qRT-PCR and normalized to EF1α. The relative expression levels of each gene in the DMSO control were set at 1. (TIF) [file pone.0117067.s003.tif]

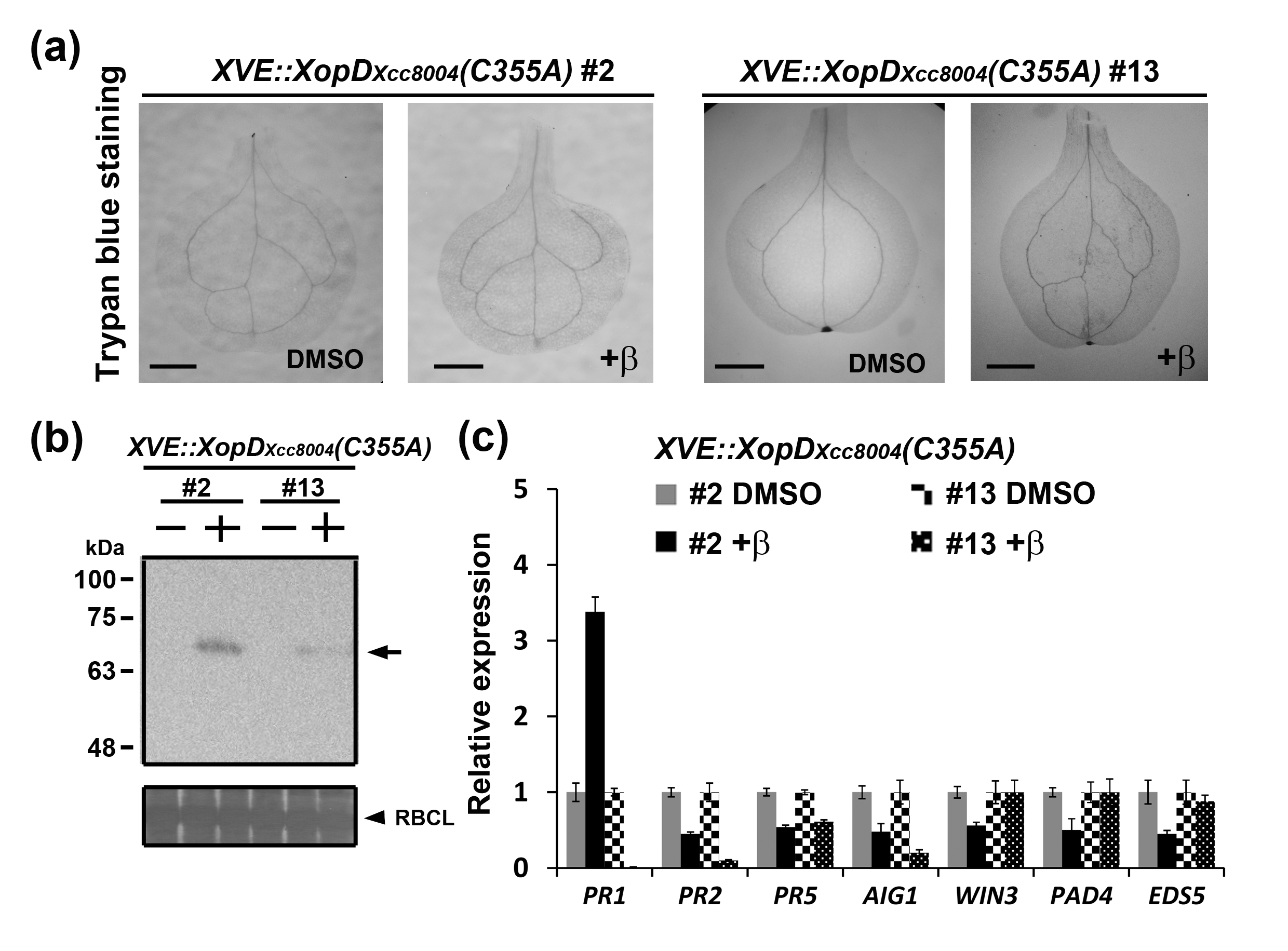

Supplement: S4 Fig — (a) Morphological examination and trypan blue staining of two-week-old leaves of Arabidopsis XVE::XopD Xcc8004 (C355A) transgenic plants. Scale bar: 1 mm. (b) Translated products of XopD Xcc8004 (C355A) were examined by western blotting using a specific antibody against XopDXcc8004 and indicated by an arrow. Rubisco large subunit (RBCL) stained with coomassie brilliant blue served as a loading control. (c) The expression levels of genes involved in the SA-mediated defense signaling network were examined by qRT-PCR and normalized to EF1α. The relative expression levels of each gene in the DMSO control were set at 1. (TIF) [file pone.0117067.s004.tif]

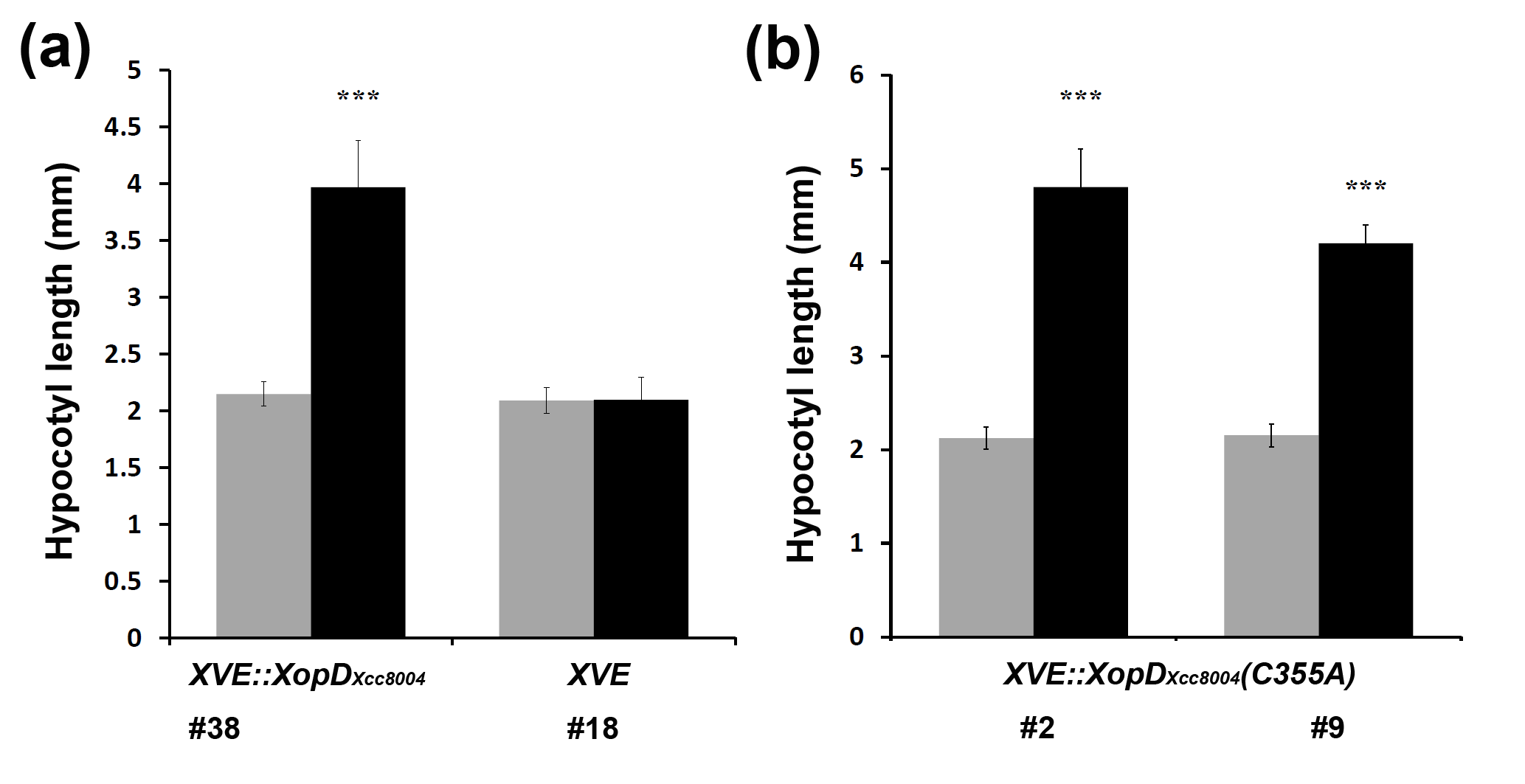

Supplement: S5 Fig — (a, b) Average hypocotyl lengths of seedlings grown on medium containing DMSO (grey bars) and 20 μM β-estradiol (black bars). Statistically significant differences were determined using one-way ANOVA (** indicates p < 0.005). (TIF) [file pone.0117067.s005.tif]
